# Geographic Distribution of Isolated Indigenous Societies in Amazonia and the Efficacy of Indigenous Territories

**DOI:** 10.1371/journal.pone.0125113

**Published:** 2015-05-13

**Authors:** Dylan C. Kesler, Robert S. Walker

**Affiliations:** 1 Fisheries and Wildlife Sciences Department, University of Missouri, Columbia, Missouri 65211, United States of America; 2 Department of Anthropology, University of Missouri, Columbia, Missouri 65211, United States of America; Midwestern University & Arizona State University, UNITED STATES

## Abstract

The headwaters of the Amazon Basin harbor most of the world’s last indigenous peoples who have limited contact with encroaching colonists. Knowledge of the geographic distribution of these isolated groups is essential to assist with the development of immediate protections for vulnerable indigenous settlements. We used remote sensing to document the locations of 28 isolated villages within the four Brazilian states of Acre, Amazonas, Roraima, and Rondônia. The sites were confirmed during previous over-flights and by image evidence of thatched-roof houses; they are estimated to host over 1,700 individuals. Locational data were used to train maximum entropy models that identified landscape and anthropogenic features associated with the occurrence of isolated indigenous villages, including elevation, proximity to streams of five different orders, proximity to roads and settlements, proximity to recent deforestation, and vegetation cover type. Isolated villages were identified at mid elevations, within 20 km of the tops of watersheds and at greater distances from existing roads and trails. We further used model results, combined with boundaries of the existing indigenous territory system that is designed to protect indigenous lands, to assess the efficacy of the existing protected area network for isolated peoples. Results indicate that existing indigenous territories encompass all of the villages we identified, and 50% of the areas with high predicted probabilities of isolated village occurrence. Our results are intended to help inform policies that can mitigate against future external threats to isolated peoples.

## Introduction

Most of the world’s last isolated tribes with limited contact to the outside world reside in greater Amazonia [1]. Their prospects for long-term isolation and survival are precarious at best, given the tragic history of previous contacts with encroaching outsiders [2–7] and the continuing onslaught of external threats that plague their existence, namely disease, displacement, and deforestation [1,2,4–12]. Government programs have been structured to protect lands for indigenous peoples in Brazil, known as indigenous territories [13]. Indigenous territories have been designated throughout the country, with particularly high densities in the Amazon Basin [13–15], but the efficacy of those programs for protecting isolated societies has not been tested, in part because of a lack of knowledge about exact locations of isolated indigenous societies. We develop a systematic methodology to estimate the distribution of isolated peoples and evaluate the utility of the existing indigenous territory system to protect them.

Locational information and population estimates are crucial for developing immediate safeguards for isolated peoples. However, there are considerable challenges to assessing demographic statistics and the actual spatial extents of their home ranges. Over-flights and chance on-the-ground encounters have thus far provided limited details [7], but these costly endeavors are invasive, in that they appear to instill reactions of fear. Previously published photographs show villagers shooting arrows at low-flying survey aircraft [16], or fleeing for cover, illustrating the level of concern that such monitoring may cause residents. Remote sensing, or the use of data gathered from distant sensors such as satellites or high elevation photographs, offers a low cost and noninvasive method for better identifying locations of isolated societies, as well as other key information on population demographics and land use. Here, we demonstrate the utility of using global information system (GIS) based remote sensing techniques to quantify demographic, spatial, and ecological characteristics of known isolated villages and to infer the locations of other, unknown isolated populations.

Similar approaches have been used in Brazil and elsewhere for the conservation of biological diversity and endangered species [17–19], which are also aimed at extending protections through governmental policies and land protection programs [20–22]. As with isolated peoples, endangered species are often rare and elusive. And, because of these characteristics, identifying occurrence/spatial distributions, ranges, key resources, and areas of importance for both isolated people and endangered species can present similar challenges. Conservation biologists have developed a number of techniques to identify and evaluate key resources and locations of species and biological systems of interest [22]. For example, reserves and reserve networks can be designed for individual species or groups of species with similar life histories [23,24], or they can be designed to protect umbrella species, which have broad ecological requirements that encompassed those of other species and communities [25]. Additionally, conservation biologists have developed tools to evaluate efficacy of conservation reserve networks to address issues associated with extractive activities and changing global conditions [26–29].

The same techniques developed and widely used to identify areas of importance for the conservation of biological diversity, and to assess the efficacy of alternative conservation policies, may have utility to cultural anthropologists and governmental policy makers attempting to provide safeguards for isolated indigenous peoples. Here, we use remote sensing data to identify the locations of isolated villages in the Amazon Basin, and to estimate the total populations of people living in each. We further use presence data and maximum entropy modeling [29–33] to develop a predictive model of the geographic distribution of isolated indigenous villages in the region. This technique uses observations collected from on-the-ground encounters, over-flights, and systematic evaluations of satellite imagery to model landscape features characteristic of areas used by isolated peoples, and then it applies that model to the entire region to derive a geographically explicit probability density function that identifies areas likely to harbor additional isolated societies. In a final analysis phase, we use a maximum entropy model, along with the boundaries of existing protected indigenous territories, to assess the effectiveness of current policy for protecting as of yet isolated societies.

## Materials and Methods

### Study Area

We studied isolated villages in the Brazilian states of Acre, Amazonas, Rondônia, and Roraima. The study area was bounded by these states because of the availability of geographic information for the country and our sampling focus on forested regions. Further, we considered only forested lands within the study area that are not part of large-scale deforestation [34] because our investigation addressed forest-dwelling groups that leave characteristic satellite imagery signatures of small slash-and-burn horticultural fields and cleared villages. The resulting study site included 154,980 km^2^ of forest in Acre, 5,873,843 km^2^ of forest in Amazonas, 237,428 km^2^ of forest in Rondônia and 224,380 km^2^ of forest in Roraima. All portions of this study were conducted using remotely collected data from satellites and high altitude photography. Thus, no specific permissions were required for field activities such as accessing lands, interacting with residents of the region, or for disturbing endangered or protected species.

### Settlement Identification

**Fig 1:**
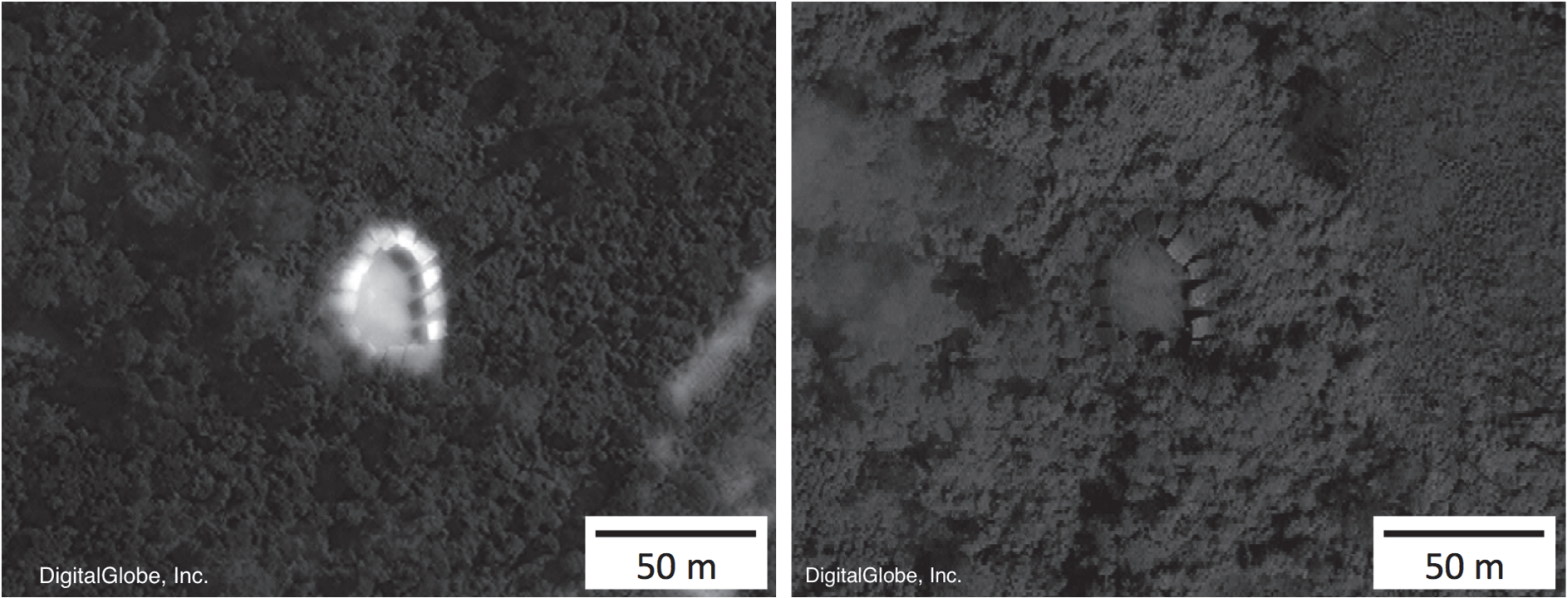
Yanomamö village. Images from an isolated Yanomamö settlement in 2012 and 2014, as identified with remote sensing. Satellite images courtesy of DigitalGlobe, Inc.

The Brazilian governmental agency FUNAI (Fundação Nacional do Índio) periodically releases information concerning approximate locations of isolated villages [1,7]. We refined these approximate locations using reports and accompanying news stories stemming from over-flights. We used published maps of recent deforestation [35] and other satellite images with high resolution (e.g., TerraServer [36] and Google Earth [37]) to visually search for forest clearings in areas with reports of isolated villages. To date, we have used this method to identify precise locations of 28 isolated communities within the study area. Further, we purchased 50-cm resolution satellite images from DigitalGlobe, Inc. (Herndon, VA, USA) for each site [38]. We then used the Image Analyst tool in the GIS (ArcMap 10.2, ESRI, Redlands, CA) to conduct an ad-hoc exploration of spectral reflectance in the images, during which we confirmed the presence of gardens, village clearings, and thatched roof dwellings indicative of human settlement (Fig 1). By manually adjusting portions of the visual spectrum displayed on the computer monitor, we were able to identify details that otherwise appeared to be washed out or uniform in composition.

Most of the isolated indigenous villages are in two hotspots on the Brazil/Peru border, one in the headwaters of the Envira River in the state of Acre (n = 7) [39] and the other in the Javari Valley Region in the state of Amazonas (n = 16) [40], most, if not all, are Pano-speaking horticultural societies [1,7,8]. We also identified coordinates of an isolated village of Yanomamö (Fig 1) in the state of Roraima and included known locations for the Hi-Merimã, Massaco, Jururei, and the lone surviving man Tanarú in the state of Rondônia. Given the sensitive nature of the exact locations of isolated peoples, and potential for such data to be used in a way that adversely affects their health and wellbeing, precise coordinates of the villages are not provided herein. However, we will reveal geographic coordinates upon receiving a request from anyone demonstrating verifiable affiliations with research organizations, governmental agencies, or other entities that can provide assurances that the information will not be redistributed or used to facilitate contact with those living in voluntary isolation.

### Population Size Estimation

A previous investigation described the relationship between population size and forest clearings, including 4 isolated villages presented herein, and reported a mean value of 9.09 individuals per cleared ha including village clearings and associated gardens [39]. We used heads-up digitization in the GIS to delineate polygons encompassing cleared areas in the other 23 isolated villages for which high resolution images are available (images were not available for one of the 28 total sites). We then multiplied the previously reported mean population density by the clearing sizes to develop estimated population sizes for each village (e.g. estimated population = *A* × 9.09 people/ha, where *A* represents area in hectares).

### Village Distribution Modeling

We used a maximum entropy algorithm implemented in MaxEnt software 3.3.3k [30–32,41] to develop a village distribution model or indigenous people living in isolation within the study area at a pixel resolution of c. 1 km^2^ (0.0083 decimal degrees per pixel; sensu [18]). Maximum entropy approaches (hereinafter “MaxEnt”) are commonly used for modeling geographic distributions of endangered or rare organisms for which presence-only (in contrast to both presence and absence) observations are available, and the approach has been shown to yield high predictive accuracy and substantial conservation application [42]. MaxEnt software attempts to identify a target probability distribution of maximum entropy, subject to constraints representing incomplete information about the target distribution [18,31,33,43,44]. When applied to the geographic presence-only data, the MaxEnt probability density model is rendered for the pixels within the study area of interest, which provides an indication of the relative occurrence rate [43]. MaxEnt approaches have been frequently applied to observations of species that are not commonly observed, or for species in regions or time periods for which systematic presence-absence surveys are not available, such as historic records.

Investigators previously demonstrated the robustness of MaxEnt approaches to data with complicated relationships between variables, and to small sample sizes [17,45–48]. Several authors have discussed potential for bias when sampling regimes are not uniform [43]. Our data were drawn from a number of sources, which left us unable to fully document the distribution of sampling effort with respect to space and variable ranges. However, unlike detections of rare endangered species that might go unnoticed by untrained observers interactions and encounters with villages of isolated peoples are much more likely to be reported, and thus to be included in our dataset. We also used visual inspections of satellite imagery to verify settlements in remote locations, which we believe offsets sampling biases in those areas. Finally, we limited the study site to only the states within which we conducted remote-sensing investigations and we removed from consideration all areas that were deforested by the year 2013 [34].

We used a series of functionally relevant landscape variables to develop a MaxEnt model of village occurrence for the study area. Transit in the Amazon Basin was historically associated with rivers and streams, and we therefore hypothesized that villages that remained isolated would be at greater distances from waterways, in lower order watersheds, and in the more elevated locations within specific regions. We also anticipated that isolated villages were likely to occur far from roads, cities, and deforested areas. Quantitative river and stream variables were derived from the World Wildlife Fund’s HydroSHED drainage direction layer and stream network layer [44]. The data originally included stream orders ranging from 1–8 on the Strahler scale [49,50]. We only considered orders 1–5 because they generally occurred throughout the study area, whereas higher orders were restricted to only lower basin areas that were also correlated with low elevation. Global land cover data were drawn from the United States Geological Survey land cover map for South America [51]. Elevation data, locations of roads, and the locations of Brazilian municipalities were drawn from digital data distributed by the Brazilian government [52,53]. We used these data to develop geographic layers representing: a) the elevation (m above mean sea level) of each pixel within the study site; b) proximity to the nearest stream of order 5 or lower, 4 or lower, 3 or lower, 2 or lower, and order 1; c) proximity to the nearest registered municipality; d) proximity to the nearest road or trail; and e) land cover.

We generated a MaxEnt village distribution model using presence-only observations of isolated indigenous villages (n = 28) and geographic variables for each pixel in the study area. The village distribution model fit was assessed by five-fold cross validation [18,32], mean area under the curve (AUC) value, and binomial probability and omission error [32,54]. Previous publications indicated that AUC values >0.75 were considered to be potentially useful [55], so we utilized the same threshold measure here to identify areas of high suitability. The relative importance of variables to village distribution models was assessed using measures of variable contribution, which were based on the increase in regularized gain added, or subtracted, by each corresponding variable during each iteration of the training algorithm [30,32].

### Efficacy of Indigenous Reserves

In an additional analysis, we used a village distribution model to assess the efficacy of established indigenous territories for protecting isolated villages, and to identify additional areas where model results indicate high probabilities of isolated village presence, but that are not currently protected. We first developed a MaxEnt model using techniques similar to those described above, except that we used all available observed village locations (full village distribution model), rather than the cross validated subsets described in the previous step.

**Fig 2:**
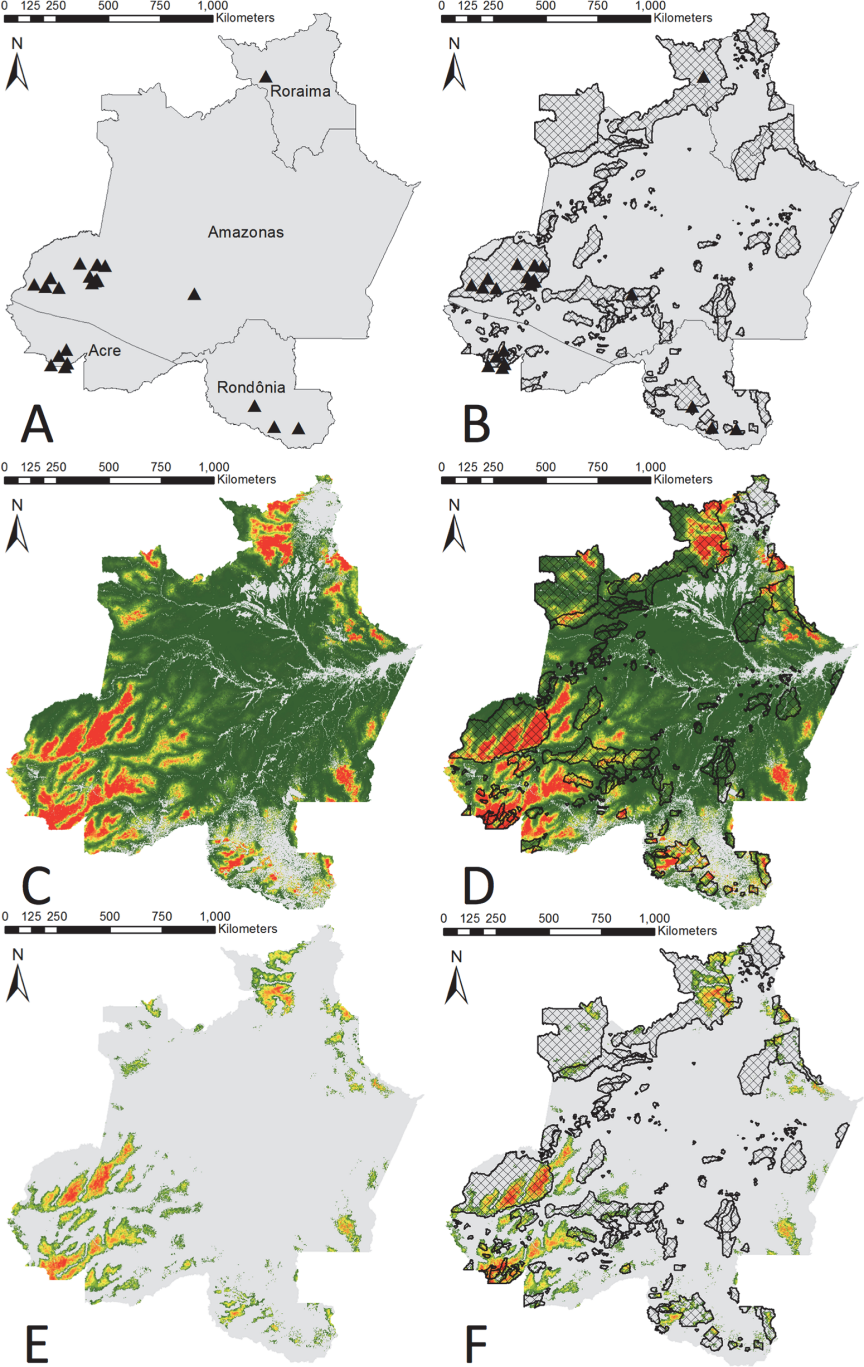
Maps of Amazon Basin study area in Brazil. Study area includes states and locations of isolated villages (triangles, panel A), and locations of isolated villages and existing indigenous reserves (crosshatch, panel B). Panel C illustrates MaxEnt model results, with red areas indicating higher probabilities of occurrence for isolated tribes. Panel D includes both MaxEnt model results and overlays of existing indigenous reserves. Panel E illustrates areas with MaxEnt occurrence probabilities that are higher than areas in which isolated tribes have actually been observed (minimum training presence), and panel F includes the overlay of existing indigenous reserves.

We used the full village distribution model to assess whether existing indigenous territories encompassed areas with high predicted occurrence values for isolated villages. We identified areas with predicted occurrence values equal to, or higher than, model values where we observed settlements of isolated tribes, or minimum training presence (MTP) areas [32,47]. All cells with a model predicted probability of occurrence greater than the MTP value from the full village distribution model were amalgamated into polygons. For each state, we report the proportion of MTP polygons within the currently existing indigenous reserves [53], and the proportion outside existing reserve boundaries. Further, we identified the locations of MTP areas that are not encompassed by existing indigenous reserve boundaries (Fig 2). We excluded deforested areas, urban areas, rivers, and streams, and then summed values for cells inside and outside the boundaries of indigenous territories within each of the four states.

## Results

We identified a total of 191.8 ha of cleared areas across 27 (high resolution imagery was not available for 1 village) isolated villages associated with higher resolution images in our four-state study area. When multiplied by previously reported population densities, results indicate that the estimated populations of villages considered here ranged from 3 to 281, with a mean of 65 and a median of 35 individuals. In total, results indicated that our work addresses approximately 1,743 individuals.

**Fig 3:**
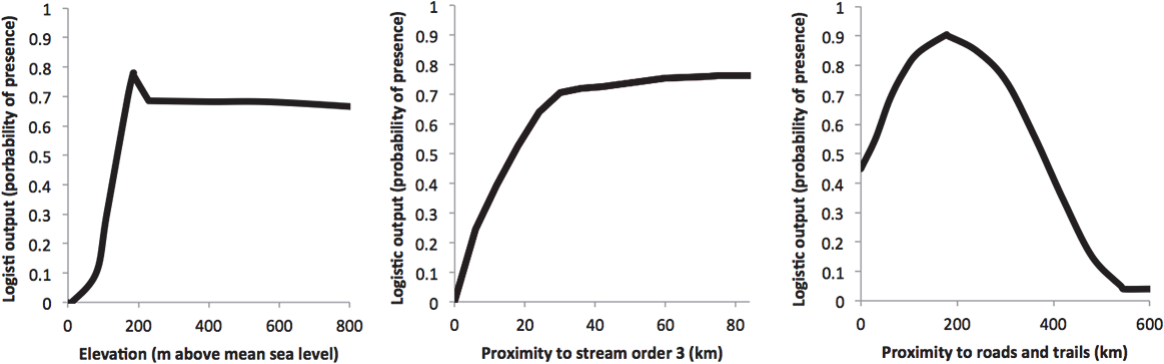
Marginal response curves. Marginal response curves for the top three variables included in the MaxEnt model of occurrence of isolated tribes in the Amazon Basin of Brazil. Plots reflect the dependence of predicted site suitability on each variable, and on dependencies induced by correlations between variables.

The village distribution model resulting from the first stage MaxEnt analysis of the 10 environmental variables identified regions of high occurrence probabilities, primarily in the southwestern portions of the study area in the states of Amazonas and Acre (Fig 2). Results further indicated strong model fit (mean AUC = 0.899; SD 0.041), so the resulting model was well above the utility threshold of 0.75. Site elevation, proximity to streams of orders 3 or higher, and proximity to roads were most influential in the model, and together the variables accounted for 72% of the model contribution (Table 1). The shape of the marginal response curves (Fig 3 and S3 Fig) for elevation indicated that village occurrence probabilities were low at the lowest elevations and that they peaked at approximately 150 m before declining again at higher elevations. Similarly, model results indicate that occurrence probabilities were low in areas close to streams of order 3 or higher, but that probabilities increased and plateaued at approximately 26 km away. Village occurrence probabilities also peaked at approximately 156 km from existing roads and trails, and then decline with greater distances. Notably, response curves for environmental variables for the village distribution model, and marginal curves created using only single variables were generally similar to the multivariate results (Fig 3).

**Table 1:** Variables and model contribution.

Variable	Dataset	Year	Model Contribution (%)
Elevation	ASTER Global Digital Elevation Model [52]	2001	40.6
Stream order 3	HydroSHED [44]	2008	21.2
Road distance	IBGE [53]	—	10.5
Stream order 5	HydroSHED [44]	2008	6.5
City distance	IBGE [53]	—	5.7
Stream order 6	HydroSHED [44]	2008	5.6
Land cover	USGS Global Land Cover Characterization [51]	2000	3.8
Deforestation	PRODES project [34]	2013	3.6
Stream order 1	HydroSHED [44]	2008	1.9
Stream order 2	HydroSHED [44]	2008	0.7

Geographic variables used for development of a settlement distribution model of isolated villages in the Brazilian Amazon Basin, and resulting percent contribution to MaxEnt model, as averaged over the 5 cross validation replicate runs. Relative percent contribution was defined as the increase in regularized gain added, or subtracted, by each variable.

In the second analysis phase, we developed a full village distribution model using all observed village locations together (S4 Fig and S1 Dataset), which was similar to the cross validated model described above. In order of importance, the top three variables were identical to those ranked in the previous phase, and they accounted for 74% of the model contribution (Table 1). Results also indicated strong model fit in the second village distribution model (AUC = 0.949; S1 and S2 Figs). The MTP value for the full village distribution model was 0.236.

Using the full village distribution model we identified areas with model occurrence values > the MTP value of 0.236, which included 266,527 km^2^ of forest (Fig 2). Of the area with occurrence probabilities > the MTP, 49.6% is incorporated into existing indigenous territories. Similarly, MTP areas already included in the indigenous territory system were characterized by a mean model value that was 0.474, whereas mean model values for MTP areas outside of reserves was 0.428 (Table 2). Large portions of Acre and southwestern Amazonas were classified by the model as MTP areas, but are not currently protected. Similarly, portions of Rondônia also had high predicted occurrence probabilities, although much of the region has already been impacted by deforestation and was thus not included in our analyses.

**Table 2:** Indigenous territory overlap with MTP area.

State	MTP Area (km^2^)	% Total MTP Area	% Total MTP Protected
Acre	57,402	22%	7%
Amazonas	141,896	53%	28%
Rondônia	22,805	9%	4%
Roraima	44,425	17%	13%

Efficacy of existing indigenous territories in the Amazon Basin for protection of isolated societies, as expressed by the area with predicted village occurrence values that were higher than the minimum training presence (MTP) value.

## Discussion

We successfully used MaxEnt methods to develop a model of the geographic distribution of isolated villages in the Amazon Basin, and we employed the model to assess the efficacy of existing indigenous territory system for protecting areas with a high probability of hosting additional isolated societies. To our knowledge, this is the first attempt to develop a quantitative model representing the likelihood of geographic and ecological occurrence of indigenous people living in isolation, or to assess the utility of existing indigenous territories for protecting those societies. Results identified strong-fitting village distribution models indicate many areas with high isolated village occurrence probabilities are currently protected, but that substantial unprotected portions of Acre and Amazonas may also harbor isolated, and as of yet undocumented, communities.

The village distribution models were strongly influenced by the elevation variable, which accounted for 43% of the full model contribution. Response curves indicated that predicted occurrence probabilities were lowest at lower elevations, although they quickly ascended to a plateau at median elevations (~180 m). Elevation variable results complement those described below for proximity to streams, in that the lowest elevation areas are also closest to the highest order streams, including the Amazon River itself (stream order 8). And the proximity of these low elevation areas to larger streams has made them accessible to explorers and outside contacts since the earliest arrival of the European explorers. However, elevation results also indicate that the likelihood of occurrence for isolated villages declines at the highest elevations in the study area, which are also the most distant from existing settlements in many cases. Indeed, the diversity of biotic communities declines at the highest elevations in the study region [56], and thus the areas likely also offer limited sustenance to human populations.

The variables associated with proximity to lower order streams also strongly influenced the village distribution models. Together, streams of orders 3, 4 and 5 accounted for approximately 32.8% of the model contribution, and the resulting response curves indicated that the probability of village occurrence was low nearby lower order streams, but that occurrence probability increased at short distances that are within a day’s walk (c. 28 km). Interestingly, 1^st^ and 2^nd^ order streams contributed only slightly (1.4%) to the village distribution model, perhaps because they are too small to be useful for transportation, and because of their ubiquity. In the Amazon Basin, streams of second and third orders are ephemeral and occasionally dry. For example, video documentation of the Envira River in Acre, where it crosses into Peru (2^nd^ order) illustrates that during the peak dry season residents were forced to walk their canoes.

Finally, the variable associated with proximity to documented roads and trails contributed approximately 11.1% to the full village distribution model—the response curve illustrates that occurrence probabilities are greatest at approximately 150 km. Roads and trails have been established at a rapid rate in recent years [18], although they remain low in density in most forested regions of the Amazon Basin. And as one might expect, increased proximity to roads likely increases opportunities for contact and enhances the chances of village documentation. However, transportation, and thus connectivity with the outside world, was historically based on stream and river networks in the Amazon basin, which may explain why streams are so strongly represented in the model and why roads are secondary.

Other variables associated with established settlements, proximity to deforestation, and land cover did not contribute markedly to the village distribution model (total contribution 13%). A likely reason for the low impact of these metrics on the performance of the village distribution models is that the variables are correlated with other variables that were more influential. For example, it is difficult to imagine that proximity to towns and settlements does not relate to village isolation, but rather that the relationship is more fully addressed through variables associated with roads and trails, or streams. Alternatively, processes such as logging and deforestation may move so quickly into some locations that isolated people become surrounded on all sides and cannot move as is the case with the lone surviving man Tanarú and several recent contacts in Rondônia [1,7]. Or perhaps some regions are changing so quickly that datasets representing deforestation and change may not be temporally representative.

Our results further indicate that a substantial proportion of MTP areas—those with highest predicted probabilities of occurrence of isolated villages—are already encircled by the current indigenous territory system. Nonetheless, numerous areas in Acre and Amazonas with high predicted occurrence probabilities remain outside the established boundaries of currently protected areas. Predicted probabilities of village occurrence for MTP areas outside the existing indigenous territory system were generally lower than areas inside indigenous territories, however, this is likely due at least in part to the fact that all of our documented locations are within protected territories. Thresholds set on MTP values have previously been identified as being conservative, especially when limited numbers of observations are used for training data [47]. We intentionally used a conservative approach to avoid over-broad predications, but for the same reason we caution that additional areas in the Amazon Basin, with model values lower than the MTP, also have substantial potential for hosting isolated villages. Further work is needed to confirm the potential locations of isolated villages outside of these areas using our MaxEnt model as a guide and perhaps warranting the extension of forthcoming protection to these regions.

## Conclusions

We recommend additional investment in the identification and protection of isolated communities in the Amazon Basin. Remote sensing and maximum entropy tools described herein provide guidance that might help target analytical approaches that will allow monitoring and protections for vulnerable groups of people living in relative isolation, without the substantial disturbances associated with over-flights or on-the-ground encounters. Similarly, these methods may be used in other regions—through the development of additional models or by applying our model and validating results. Finally, the extension of additional aspects of conservation reserve network design theory [26,29] may have additional utility to those attempting to provide protections to isolated indigenous people.

## Supporting Information

S1 Fig Omission rate and predicted area. Omission rate and predicted area for the full village distribution model of isolated village occurrence in the Amazon Basin of Brazil, as a function of the cumulative threshold, which is calculated based on the training presence records.
(TIFF)

S2 Fig Receiver operating characteristic (ROC) curve. ROC curve for the full village distribution model of isolated village occurrence in the Amazon Basin of Brazil.
(TIFF)

S3 Fig Marginal response curves. Marginal response curves for variables included in the full village distribution model of isolated village occurrence in the Amazon Basin of Brazil. The curves illustrate how the logistic prediction changes as each environmental variable is varied, keeping all other environmental variables at their average sample value.
(TIFF)

S4 Fig Full MaxEnt model. Full village distribution model, as presented by program Maxent. From blue to red, colors indicate increasing probability of occurrence of isolated villages in the Amazon basin. White boxes represent training data based on known village locations.
(TIFF)

S1 Dataset Full MaxEnt model in KMZ format. Google Earth KMZ layer file with graphic representation of the full MaxEnt model.
(ZIP)
