# Supplementary figures and images for: Geographic Distribution of Isolated Indigenous Societies in Amazonia and the Efficacy of Indigenous Territories
Source: PLoS One. 2015 May 13;10(5):e0125113. doi: 10.1371/journal.pone.0125113 (PMC4430527; doi:10.1371/journal.pone.0125113)

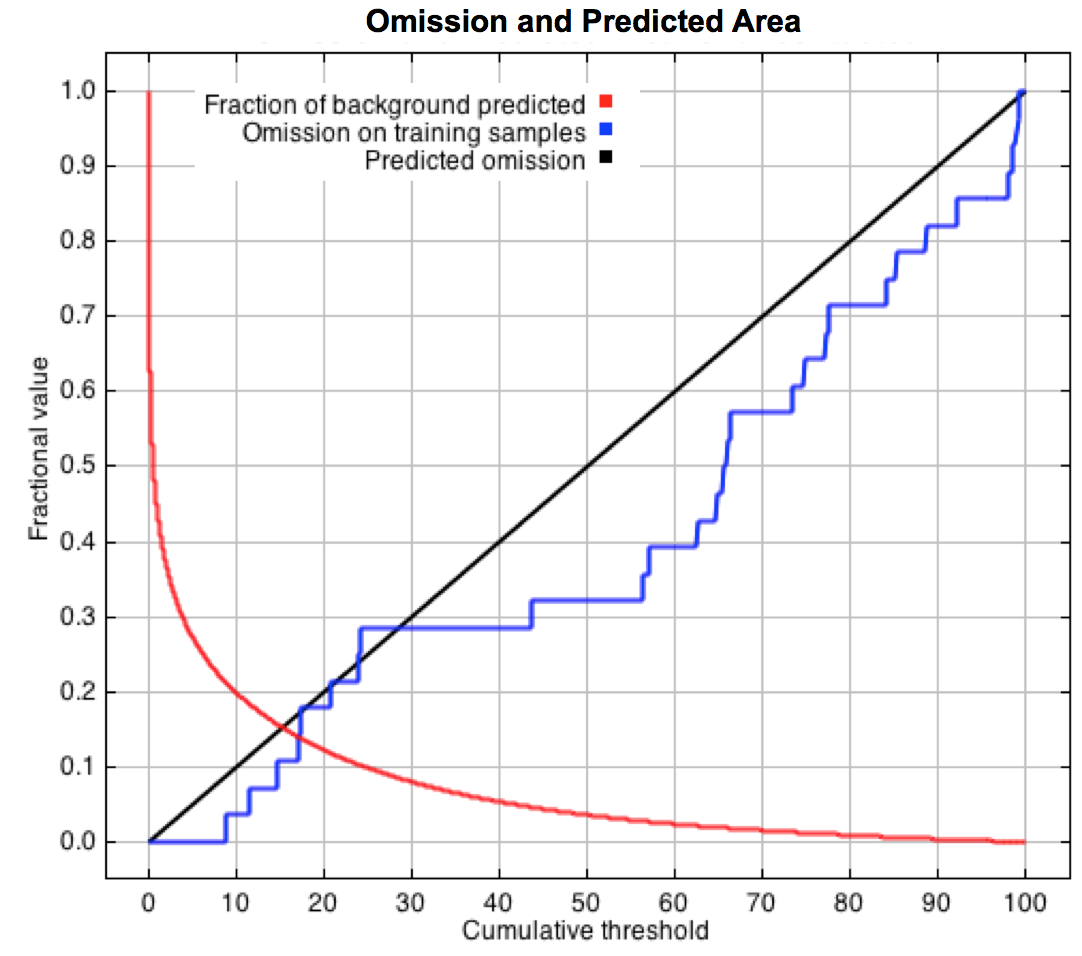

Supplement: S1 Fig — Omission rate and predicted area for the full village distribution model of isolated village occurrence in the Amazon Basin of Brazil, as a function of the cumulative threshold, which is calculated based on the training presence records. (TIFF) [file pone.0125113.s001.tiff]

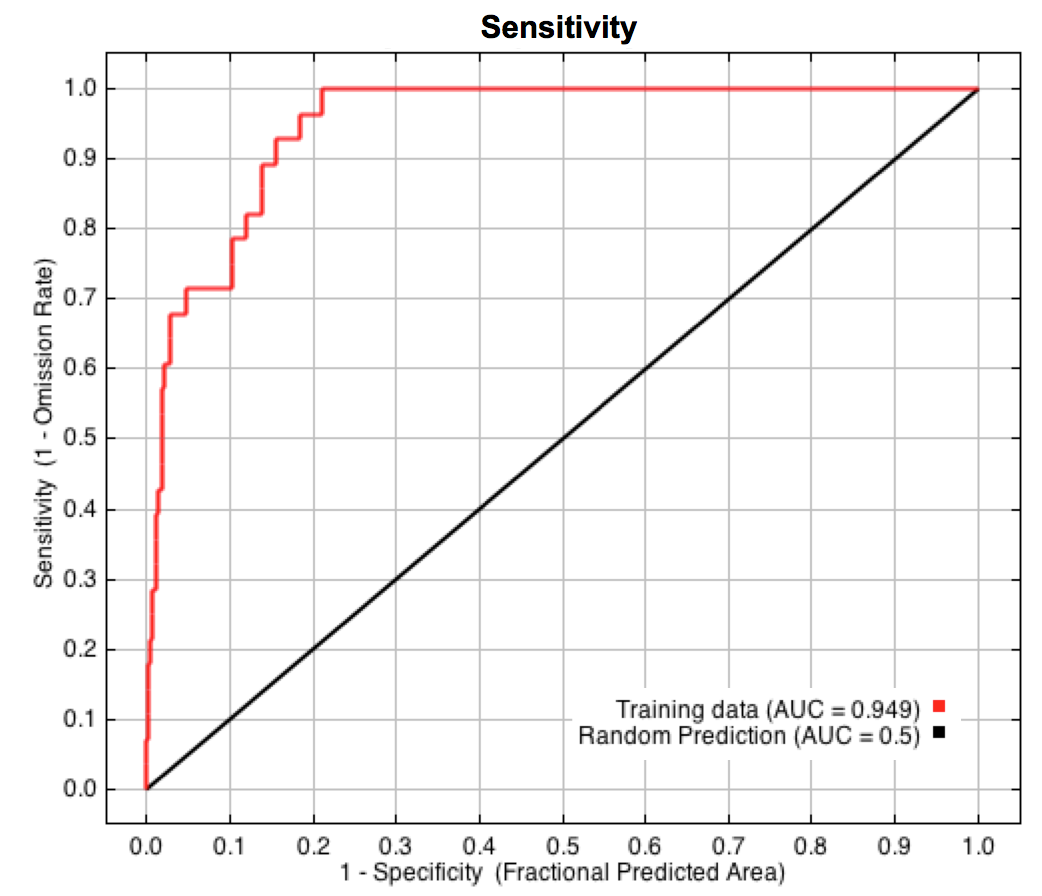

Supplement: S2 Fig — ROC curve for the full village distribution model of isolated village occurrence in the Amazon Basin of Brazil. (TIFF) [file pone.0125113.s002.tiff]

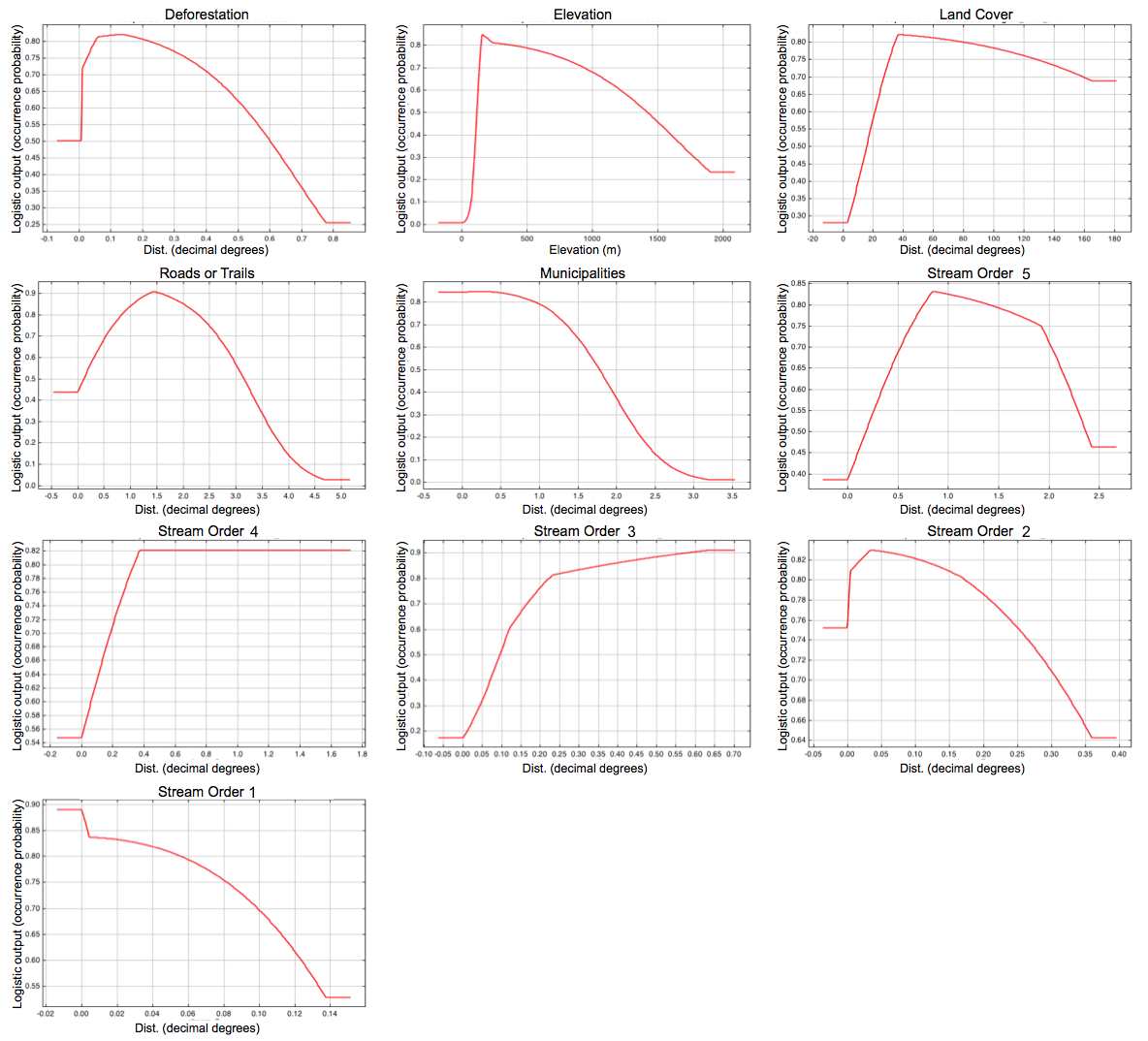

Supplement: S3 Fig — Marginal response curves for variables included in the full village distribution model of isolated village occurrence in the Amazon Basin of Brazil. The curves illustrate how the logistic prediction changes as each environmental variable is varied, keeping all other environmental variables at their average sample value. (TIFF) [file pone.0125113.s003.tiff]

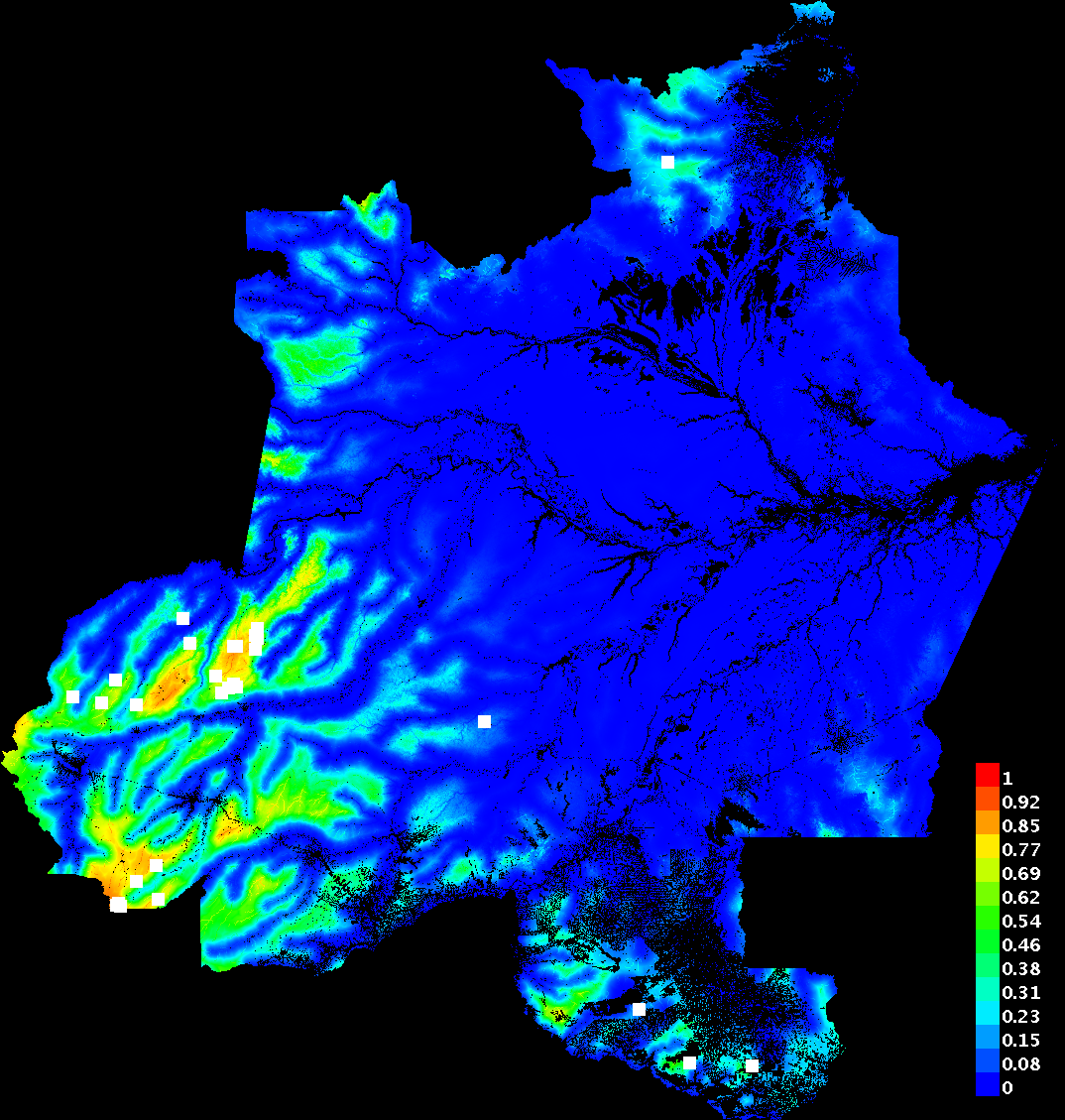

Supplement: S4 Fig — Full village distribution model, as presented by program Maxent. From blue to red, colors indicate increasing probability of occurrence of isolated villages in the Amazon basin. White boxes represent training data based on known village locations. (TIFF) [file pone.0125113.s004.tiff]
